# The Ecological Role of Type Three Secretion Systems in the Interaction of Bacteria with Fungi in Soil and Related Habitats Is Diverse and Context-Dependent

**DOI:** 10.3389/fmicb.2017.00038

**Published:** 2017-01-31

**Authors:** Rashid Nazir, Sylvie Mazurier, Pu Yang, Philippe Lemanceau, Jan Dirk van Elsas

**Affiliations:** ^1^Department of Environmental Sciences, COMSATS Institute of Information TechnologyAbbottabad, Pakistan; ^2^Department of Soil Environmental Science, Research Centre for Eco-environmental Sciences – Chinese Academy of SciencesBeijing, China; ^3^Agroécologie, AgroSup Dijon, Institut National de la Recherche Agronomique, Université Bourgogne Franche-ComtéDijon, France; ^4^Department of Microbial Ecology, GELIFES, University of GroningenGroningen, Netherlands

**Keywords:** bacteria, fungi, interactions, microbial ecology, soil, type three secretion system, mycorrhiza

## Abstract

Bacteria and fungi constitute important organisms in many ecosystems, in particular terrestrial ones. Both organismal groups contribute significantly to biogeochemical cycling processes. Ecological theory postulates that bacteria capable of receiving benefits from host fungi are likely to evolve efficient association strategies. The purpose of this review is to examine the mechanisms that underpin the bacterial interactions with fungi in soil and other systems, with special focus on the type III secretion system (T3SS). Starting with a brief description of the versatility of the T3SS as an interaction system with diverse eukaryotic hosts, we subsequently examine the recent advances made in our understanding of its contribution to interactions with soil fungi. The analysis used data sets ranging from circumstantial evidence to gene-knockout-based experimental data. The initial finding that the abundance of T3SSs in microbiomes is often enhanced in fungal-affected habitats like the mycosphere and the mycorrhizosphere is now substantiated with in-depth knowledge of the specific systems involved. Different fungal–interactive bacteria, in positive or negative associations with partner fungi, harbor and express T3SSs, with different ecological outcomes. In some particular cases, bacterial T3SSs have been shown to modulate the physiology of its fungal partner, affecting its ecological characteristics and consequently shaping its own habitat. Overall, the analyses of the collective data set revealed that diverse T3SSs have assumed diverse roles in the interactions of bacteria with host fungi, as driven by ecological and evolutionary niche requirements.

## Introduction

Bacteria can interact closely with eukaryotic hosts, as recently illustrated in studies on the core microbiomes that are associated with plants and supported by the proposal that plants and their associated microorganisms may be considered as “superorganisms” denoted holobionts (Vandenkoornhuyse et al., 2015). Interactions between the microbial and plant components of this holobiont are thought to be tuned by molecular communication, especially that relying on systems such as the type III secretion system (Lemanceau et al., 2016). Although we still understand little about the holobiont of fungi, one might propose a similar concept for fungi in soil and related habitats. This field of research is important since bacteria and fungi are essential contributors to biogeochemical cycles in soil. They are also important for plant nutrition and health. Hence, molecular communications between bacterial and fungal communities are highly relevant for sustainable soil management (Lemanceau et al., 2016). Soil fungi have recently been implicated in the translocation of a large fraction of the plant photosynthates via hyphal networks into the soil (Klein et al., 2016). In soil, a suite of bacteria is known to interact nutritionally with fungal counterparts. The fungi often perform key steps in the breakdown of complex organic materials, yielding small molecules which are then further decomposed by bacteria that occur in the same habitat. The latter may contribute to nutrient provision to plants as well, e.g., performing important steps of the nitrogen cycle, such as nitrogen fixation (Nelson and Sadowsky, 2015) or phosphate solubilization (Nazir et al., 2016). Due to their mycelial way of growth, fungi can form true networks of interconnected hyphae in the soil as well as in the mycorrhizosphere and endosphere. These mycelial networks constitute an “evolutionary playroom” for soil bacteria, as they may have ‘grabbed the opportunities’ offered to them by these networks (Zhang et al., 2014). Thus, bacteria and fungi together, given their different functional roles, serve as the basis of soil food webs (Rudnick et al., 2015). Along with this role, bacteria and fungi mediate the growth, development and health of their host plants (Heydari and Pessarakli, 2010).

Concerning the mutual interactions, fungi clearly impact the composition of bacterial communities in their sphere of influence (the mycosphere; Warmink and van Elsas, 2008). Scheublin et al. (2010) also found that bacterial communities attached to the hyphae of *Glomus intraradices* and *G. proliferum* had undergone strong selection. These belonged mostly to *Oxalobacteraceae* and differed from the bacteria attached to non-hyphal plant roots or glass wool substrate. These and other studies suggested that fungal hyphae tend to drive associations with specific bacterial groups. In terms of ecological outcomes, the interactions between bacteria and fungi vary from symbiotic and mutually beneficial (Partida-Martinez et al., 2007) to deleterious, in which the viability of one of the associates is affected (Scherlach et al., 2013). For instance, bacteria belonging to the genus *Collimonas* exhibit antifungal activity and are able to grow at the expense of the living fungi. In a key study, *Collimonas fungivorans* was shown to grow well and inhibit the hyphal spread of *Aspergillus niger* when the organisms were confronted with each other (Mela et al., 2011). In contrast, other bacterial–fungal associations have evolved into obligately synergistic ones. For instance, in the association of the bacterium *Burkholderia rhizoxinica* with *Rhizopus microsporus*, it provides toxins to its host fungus, allowing the invasion by the latter of rice seedlings for mutualistic nutrient acquisition (Schmitt et al., 2008). In return, the bacterium acquires a place to live, in this case inside the fungal host. On another notice, soil-exploring fungi (crossing air gaps) can help bacteria to move from one microhabitat in soil to another one (Kohlmeier et al., 2005; Furuno et al., 2010, 2012a,b; Warmink et al., 2011; Bravo et al., 2013). Remarkably, along with nutrition (glycerol) and access to new habitats [migration with the fungus *Lyophyllum* sp. strain Karsten (Nazir et al., 2012)], *Burkholderia terrae* BS001 was shown to affect fungal physiology via inhibition of mushroom formation (Nazir et al., 2013c). During another mutualistic association, i.e., *Pseudomonas putida* with *Morchella crassipes*, the bacterium was found to gain advantage through dispersal and rearing, while the fungus did so through additional carbon source acquisition and enhanced stress resistance (Pion et al., 2013). On the basis of these examples, we posit that fungal-influenced microhabitats that spur the development of mycelium-associated bacteria are of utmost importance in the terrestrial ecosystem.

In the light of the aforementioned scenarios of interactions, a major challenge is to identify the microbial traits that are involved in these interactions. Miransari (2011) summarized the interactions of soil fungi and bacteria, including the binding of soil bacteria to fungal spores followed by molecule injection, volatile compound production and degradation of fungal cell walls. Haq and van Elsas (2015) expanded these concepts and proposed a stepwise progressive interaction of bacteria with their fungal hosts. Such stepwise mechanisms have obvious consequences for microbial gene expression and performance, and consequently drive the ecological interactions between bacterial and fungal partners in a dynamic and temporally explicit manner. Bharadwaj et al. (2008) reported a set of 10 different bacterial strains isolated from fungal spores to be multifunctional in the mycorrhizosphere, showing that diverse extracellular enzymes and bioactive compounds were at the basis of this multifunctionality. Together with evidence from Haq et al. (2014; 2017), this finding hinted at the importance of bacterial protein secretion systems for survival in fungal-associated habitats. Thus, we postulate that an important molecule ‘release’ system is often operational at (mycorrhizal) fungi, resulting in exudates that attract particular bacteria from their vicinity. Therefore, among the possible mechanisms mediating bacterial–fungal interactions (BFIs) in this habitat, attention should be given to bacterial secretion systems. In particular, bacterial type three secretion systems T3SS, which for a long time were only considered to constitute virulence determinants of Gram-negative bacteria, may play roles in the modulation of bacterial-fungal interactions (BFI) in soil and thus soil functioning. In this review, we critically examine the literature on this topic, with the following specific objectives:

(1) Consideration of the evolution of the T3SS and its connection with interactions with particular hosts,
(2) Evaluation of the (potential) role of the T3SS in BFI in soil and soil-related habitats,
(3) Outlook and identification of future research directions.

### T3SSs – Origin, Evolution, and Divergence

Type III secretion systems are intricate proteinaceous systems which span two membranes in Gram-negative bacteria, thus offering an outlet from the cytoplasm to the outside milieu. The T3SS machinery is composed of 20-odd conserved proteins (up to 30 in some bacteria), forming a structure containing an elaborate base, an inner rod and a needle (Tseng et al., 2009). Moreover, so-called effector proteins are often encoded by a T3SS gene region, which – upon extrusion via the T3SS, may modulate the physiology of recipient cells. Phylogenetic analyses confirm that often T3SS proteins from one bacterial group are related to the ones from other bacteria, rather than being novel and different (Silva et al., 2013). The analyses demonstrated that T3SSs originate from the flagellum via recruitment of a part for the evolution of protein delivery functions and secretins. The descendants of an intermediate ancestral form still exist in the *Myxococcales*. These lack essential elements for motility, while containing a subset of T3SS features (Abby and Rocha, 2012). Thus, because of the great resemblance of the T3SS to the bacterial flagellar system, the term NF (non-flagellar) – T3SS has been recently coined (Abby and Rocha, 2012) to delineate all T3SSs with dedicated secretion roles. From here, we will only deal with this NF-T3SS, which is denoted, for the reasons of simplicity, as the ‘T3SS.’ The T3SS proteins can basically be grouped into three categories:

(1) Structural proteins, building the base, inner rod and needle structures;
(2) Effector proteins, which are secreted to outside of the cell and probably into a eukaryotic host cell;
(3) Chaperones, which bind the effectors in the bacterial cytoplasm, protecting them from aggregation and degradation and directing them toward the needle complex.

With respect to the T3SS nomenclature, the literature contains various abbreviations that have been given independently to series of proteins in each organism. Some proteins that were initially discovered independently in different bacteria have later been shown to be homologous (Wang et al., 2012), but the historical names have often been kept. For example, the proteins SicA, IpgC, and SycD are homologs of each other (described for *Salmonella, Shigella*, and *Yersinia*, respectively) and these and other names have persisted in the literature. Moreover, a capital letter at the end of a T3SS protein name indicates the order of discovery or the physical order of appearance of the particular protein-encoding gene in an operon, e.g., IpaA, IpaB, IpaC. Otherwise, numbers denote the molecular weight of the protein in kDa, e.g., Spa9, Spa47. Thus, there are still several names and coding systems in use for a similar or identical gene, although a common nomenclature, i.e., the *sct* (secretion and cellular translocation) system, has long been proposed (Hueck, 1998). We advocate that this latter system should be widely adopted for all genes and proteins of the T3SSs, thus harmonizing the nomenclature. However, for reasons of simplicity we still use the gene and protein names as they appear in the literature.

**FIGURE 1:**
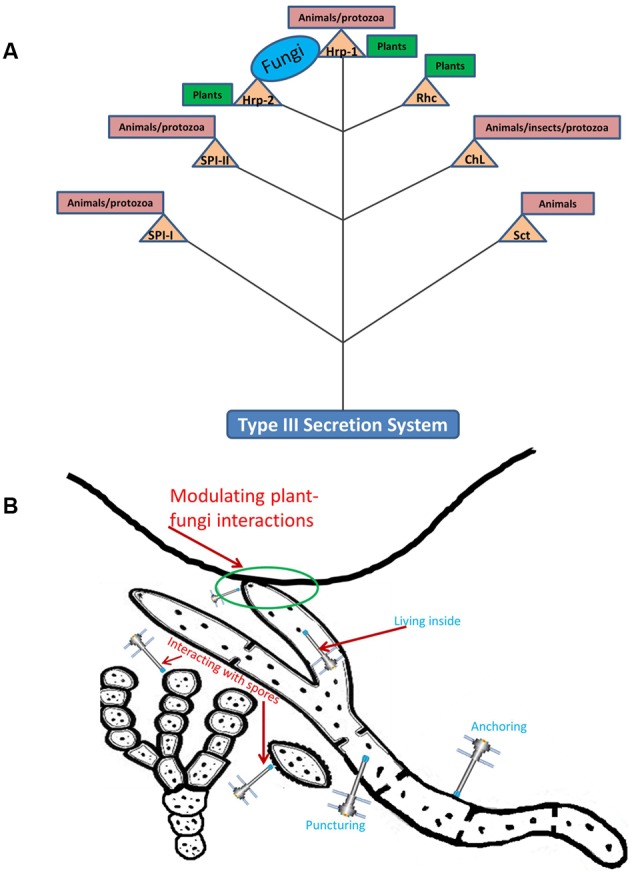
**Schematic representation of different bacterial type III secretion systems (T3SS). (A)** Illustrating the interactive roles of T3SS with potential eukaryotic hosts. **(B)** Depicting the potential role(s) of T3SS during interactions with fungi, on their surfaces, inside the hyphae and with spores. Triangles: different T3SS family; circles, rectangles: eukaryotic hosts. Branching: no evolutionary meaning implicated. Rhc, *Rhizobia* conserved; ChL, *Chlamydiales*; Sct, secretion and cellular translocation; SPI, *Salmonella* Pathogenicity Island; Hrp, hypersensitive response and pathogenicity. Thick solid line on top of **(B)** represents a plant root system demonstrating the mycorrhizal interaction.

Being versatile in ecological functioning, the T3SS has evolved, over evolutionary time, into seven different families (illustrated in **Figure 1A**), denoted as Ysc, Hrp1, Hrp2, SPI-1, SPI-2, RhC (*Rhizobiales*) and ChL (*Chlamydiales)* (Pallen et al., 2005; Troisfontaines and Cornelis, 2005). These different types of T3SSs are grossly related to the ecological roles their hosts play when interacting with eukaryotic hosts (Abby et al., 2014), as discussed in the following. *Grosso modo*, obligatorily intracellular bacteria that live in animals, insects and protozoa harbor the ChL-type T3SS, whereas the SPI-1, SPI-2, and Ysc systems are mostly present in animal- and protozoan-associated bacteria. T3SSs of the Hrp1 and Hrp2 families are mainly confined to plant pathogens, while the RhC type is present in plant-interactive rhizobiales (Tampakaki, 2014) and pseudomonads like *Pseudomonas syringae* (Gazi et al., 2012; Loper et al., 2012). Some bacteria may even harbor more than one T3SS, e.g., SPI-1 and SPI-2 T3SSs are present in the human pathogen *Salmonella typhimurium.* These two T3SSs (Tseng et al., 2009) perform different roles, i.e., facilitating biofilm formation (Jennings et al., 2012) and survival in amoeboid cells (Bleasdale et al., 2009), respectively. The ecological roles of the different T3SSs are further explored in the next section(s).

## Bacterial T3SSs Have Different Roles in Interactions With Eukaryotic Hosts

### Overall Description of Roles

Type III secretion systems have been shown to be associated with diverse functions in different bacterial species (**Table 1**). Thus, a T3SS was found to mediate the cell cycle of a eukaryotic host with which the T3SS-containing bacterium interacts. In one example, during co-culturing of enteropathogenic *Escherichia coli* (EPEC) and human epithelial (HeLa) cells, a ‘cycle inhibiting factor’ (Cif; a T3SS effector) was found to assist the blocking of the HeLa cell cycle in its early phases, i.e., DNA replication (Samba-Louaka et al., 2009). Often, T3SSs support, in a generic sense, the invasion of host cells and the release of nutrients from these (Dale et al., 2001; Ochman and Moran, 2001; Moreira et al., 2006; Perrett and Zhou, 2013). Thus, they promote the interaction of particular bacteria (pathogens as well as symbionts) with their hosts (Coombes, 2009). Remarkably, T3SSs were found to play roles in the lifestyles of (1) nitrogen-fixing rhizobial mutualists of plants, (2) *Sodalis glossinidius*, the mutualist of the tsetse fly, (3) the nematode mutualist *Photorhabdus luminescens* and (4) the human commensal *Pantoea agglomerans* (Dale et al., 2001; Cornelis, 2006; Tseng et al., 2009; Nelson and Sadowsky, 2015).

**Table 1:** Different environmental bacteria, in the context of their ecological role(s) with eukaryotic Hosts, as mediated by type III secretion systems.

Bacterial partner	Eukaryotic host	Bacterial microhabitat^∗^	Interaction with host	Ecological role of T3SS	Reference
*Erwinia chrysanthemi*	Mammal	Diverse	Parasitic	Aggregative multicellular behavior	Yap et al., 2005
*Escherichia coli*	Mammal, Plant	Diverse	Parasitic/commensal	Adhesion and biofilm formation; colonization/inactivation of death domain; attachment to leaves	Moreira et al., 2006; Shaw et al., 2008; Li et al., 2013
*Salmonella enterica*	Amoeba	Diverse	Parasitic/commensal	Survival in amoeba; biofilm formation and cell clumps; block exocytosis	Bleasdale et al., 2009; Jennings et al., 2012; Perrett and Zhou, 2013
*Sodalis glossinidius*	Tsetse fly	Intracellular	Mutualistic	Enter into the host cell	Dale et al., 2001; Ochman and Moran, 2001
*Pseudomonas fluorescens* F113	Amoeba, Plant	^∗∗^Mycorrhizosphere	Mutualistic to plant, parasitic to amoeba	PGPR; resistance to amoeboid grazing	Barret et al., 2013b
*Rhizobium* sp. NGR234	Plant	Rhizosphere	Mutualistic	Nodule formation	Viprey et al., 1998
*Burkholderia rhizoxinica*	*Rhizopus microsporus*	Fungal endosphere	Mutualistic	Successful endomycotic life style	Lackner et al., 2011a,b
*Burkholderia terrae*	*Lyophyllum* sp. *Karsten*	Mycosphere	Mutualistic	Fungal attachment and migration impairment	Haq et al., 2016; Yang et al., 2016
*P. fluorescens* KD	*Pythium ultimum*	Mycorrhizosphere	Parasitic	Reduced activity of pectinase, polygalacturonase (a pathogenicity factor)	Rezzonico et al., 2005
*S. enterica*	*Candida albicans*	Diverse	Parasitic	Fungal killing	Tampakakis et al., 2009; Kim and Mylonakis, 2011
*P. fluorescens* BBc6R8	Plant-EMF(*Douglas fir* – *Laccaria bicolor*)	Mycorrhizosphere	Mutualistic	Promote ectomycorrhization	Cusano et al., 2011
*P. fluorescens* C7R12	Plant–AMF (*M. truncatula* mycorrhizas)	Mycorrhizosphere	Mutualistic	Promote arbuscular endomycorrhization	Viollet et al., 2016

*^∗^The origin of the strain(s) and/or the environment in which the relevant study has been performed, please refer to the appropriate reference for specific details.*

*^∗∗^Generally rhizosphere is mycorrhizosphere, so we did not make specific distinction; please refer to the relavent study for specific information. EMF, ectomycorrhizal fungus; AMF, arbuscular mycorrhizal fungus; PGPR, plant growth promoting rhizobacterium.*

Considering the foregoing, we argue that the T3SS constitutes a generic hallmark of a broad array of Gram-negative bacterium–eukaryote interactions, rather than just of pathogenesis (Tseng et al., 2009). It appears to endow the respective bacterial cells with an organelle that enables these to successfully occupy the niches that, following effector injection, are provided by eukaryotic hosts (He et al., 2004). Coombes (2009) suggested that diverse and multiple effector proteins translocated from T3SS-positive bacteria to their respective hosts provide unique opportunities to modulate the physiologies of the latter in diverse manners. Moreover, the specific ratios of these secreted effector proteins were suggested to determine the outcomes of host colonization and modulation processes (Coombes, 2009). On the basis of their molecular ‘playground,’ T3SSs are thus vital components of diverse ecological functions.

### Animal–Pathogenic Bacteria

With respect to the T3SSs found in animal-associated bacteria, several striking observations have been made. First, these T3SSs harbor a rather flexible and small needle (He et al., 2004). These T3SSs endow their hosts with a diverse range of traits. For instance, the *espA* (a gene of the T3SS machinery) encoded protein (syn: LcrV, IpaD, SipD) – next to pili – is involved in biofilm formation by EPEC (Moreira et al., 2006). Here, the T3SS effector NleB has recently been reported to inactivate ‘death domains’ (related to apoptosis) in several host proteins. It contains an *N*-acetylglucosamine (GlcNAc) transferase activity which specifically modifies a conserved arginine in these death domains. This activity was required for colonization of mice by EPEC (Li et al., 2013). Moreover, the replication of *Salmonella enterica* in its eukaryotic host occurs inside a *Salmonella*-containing vacuole (SCV), which is modified by bacterial effectors secreted through two T3SSs, T3SS-1 and T3SS-2. Interaction of these effector molecules with the host cell secretory pathway may provide *S. enterica* with nutrients, contributing membrane material necessary for SCV biogenesis, altering antibacterial peptide/protein secretion or manipulating cell surface proteins that are important in the host response to bacterial infection (Perrett and Zhou, 2013). Lara-Tejero and Galan (2009) discovered that the protein translocases SipB, SipC, and SipD of the SPI-1 type T3SS of *S. enterica* serovar Typhimurium are required for the intimate association of the bacterium with host cells. SipD was present on the bacterial surface prior to contact with the host cells. In contrast, SipB and SipC were detected at the bacterial surface only following the contact with the target cell (Lara-Tejero and Galan, 2009).

### Plant–Pathogenic Bacteria

Type III secretion system genes have been found in almost all Gram-negative plant–pathogenic bacteria, such as *Erwinia* spp., *Xanthomonas* spp., *Pseudomonas syringae*, and *Ralstonia solanacearum.* This finding provides evidence for a central role of these secretion systems in diverse bacterium-plant interactions (Alfano and Collmer, 2004; He et al., 2004; Büttner and Bonas, 2006). The T3SS machinery of these plant-interactive bacteria possesses a long and rigid needle that enables them to penetrate the plant cell wall and membrane. The available evidence suggests a strict requirement of the Hrp-type pilus for bacterial pathogens to interact with host plants (He et al., 2004). A recent functional analysis of a T3SS effector protein of *R. solanacearum*, RipTPS, with homologs in other bacterial plant pathogens, demonstrated its translocation into host plant cells. The target in plant cells, trehalose-6-phosphate, is a key signal molecule that regulates sugar status and carbon assimilation, and hence a metabolism-modulating effect of RipTPS on the plant is cogitated (Poueymiro et al., 2014).

### Host–Symbiotic Bacteria

Type III secretion systems also contribute to other host-interactive ecological roles played by bacteria. In the interaction, physical contact may be required for efficient functioning of the T3SS. A mutant of *S. glossinidius* that lacked the T3SS *invC* (*sct*N) gene could not enter tsetse fly cells, whereas the wild-type could (Dale et al., 2001). Thus a functional T3SS was essential for the mutualism between this bacterium and its host, as also shown in a complementation experiment (Ochman and Moran, 2001). The T3SS has recently also been found to promote the survival of *Vibrio parahaemolyticus* in diverse protists in a planktonic food web (Matz et al., 2011).

As another example, T3SS-positive *Bradyrhizobium* populations were preferentially enriched in the soybean rhizosphere (Mazurier et al., 2006), suggesting a role for the T3SS in the symbiosis with this host plant. Viprey et al. (1998) had already suggested that T3SS-secreted proteins, termed ‘nodulation outer proteins’ (Nops), contribute to the bacterium-plant symbiosis (Büttner and Bonas, 2006). Thus, the T3SS appears to be a key modulator of the symbiosis of *Rhizobium* with leguminous plants (Nelson and Sadowsky, 2015), via effects on nodulation (Tampakaki, 2014). Particularly, in *Rhizobium* sp. NGR234, nodule formation was found to be co-regulated with the expression of T3SS genes (Skorpil et al., 2005).

### Non-symbiotic (Free-Living) Bacteria

The T3SS may also play a role in bacteria that have long been considered as free-living. For instance, the soil dweller *P. fluorescens* F113 can form a [plant-growth promoting (PGP)] association with host plants. The strain F113 genome revealed the presence of two complete T3SSs, belonging to the Hrp1 and SPI-1 families (**Figure 1**). The SPI-1 type T3SS transcriptional activator *hil*A was induced by amoebae that were in close contact; this allowed increased bacterial survival. Indeed, a 25-fold decrease of bacterial fitness was observed for a T3SS (*spa*S gene) knock-out mutant that was challenged the same way. Hence, the SPI-1 type T3SS enhances the resistance of *P. fluorescens* to amoeboid grazing (Barret et al., 2013b). This T3SS type has been found more broadly in the genus *Pseudomonas* (Mazurier et al., 2004, 2015). Supporting the contention of a role in grazing defense was the finding of Bleasdale et al. (2009), who reported that the SPI-2 type T3SS is essential for the survival of, in this case, *S. enterica* in free-living amoebae. In addition, enterohemorrhagic *E. coli* (EHEC) has been reported to use the T3SS needle as an anchor for attachment to plant leaves (Shaw et al., 2008), thus allowing its exploration of the plant-associated microhabitat. Furthermore, a T3SS was found to be required for aggregative multicellular behavior by *Erwinia chrysanthemi* (Yap et al., 2005), providing an ecologically relevant behavioral asset to this organism. In line with this, Jennings et al. (2012) evaluated the role of the SPI-1 type T3SS-1 in an *S. Typhimurium* biofilm and cell clump formation in different media. The biofilms and cell clumps were associated with the SPI-1 T3SS-secreted proteins SipA, SipB, SipC, SopB, SopE, and SptP. However, mutations in the genes *bcs*A, *csg*BA, and *bap*A (essential for biofilms) did not affect the biofilm (Jennings et al., 2012). Abby et al. (2014) identified presumably plant-interactive proteins encoded by T3SS-like genes in microbiomes associated with the alga *Ostreococcus tauri. Flavobacterium* was the most ubiquitous bacterial group (present in 10 out of 13 *O. tauri* cultures). In six of the 13 microbiome metagenomes, putative T3SSs (Hrp1 and RhC types) were detected. On the basis of their findings, the authors posited that the T3SS likely plays a role in the interactions of bacteria with *O. tauri* (Abby et al., 2014).

Finally, T3SS genes were detected in clinical as well as environmental *Vibrio cholerae* isolates (Morita et al., 2013), hinting at diverse ecological roles. Collectively, one gets a picture of the great versatility in the ecological roles that T3SSs have taken in bacteria, spanning dedicated roles in pathogenicity as well as symbiosis on plant and animal hosts, saprophytic and ecological fitness (viz. defense against protozoan attacks, biofilm formation).

## Evidence for the Involvement of T3SSs in Bacterial–Fungal Interactions

### Evidence from Direct Molecular Studies

The effects of fungal hyphae on the T3SS distribution (**Figure 1B**) in the mycosphere have been assessed using direct DNA-based approaches. Thus, a *hrc*R (syn. *sct*R) – based PCR-DGGE system was developed to evaluate the diversity of the T3SS in the mycosphere versus bulk soil (Warmink and van Elsas, 2008). This initial culture-independent analysis showed the differential selection of *hrc*R gene types in the mycosphere of the ectomycorrhizal fungus *Laccaria proxima* compared to the respective bulk soil (Warmink and van Elsas, 2008). In a later study, the abundance of the specific *B. terrae* BS001 *hrc*R (syn. *sct*R) gene, was found to be very low in soil 15 days after inoculation with a bacterial community, in the absence of fungi. In contrast, in the same study, a selection of specific T3SS types by the mycosphere of *Lyophyllum* sp. strain Karsten was revealed. Colonization by *Lyophyllum* sp. strain Karsten thus significantly enhances the abundance of the *B. terrae* BS001 – specific *hrc*R (syn. *sct*R) type gene, as was found in four different pre-sterilized soils (Nazir, 2012; Nazir et al., 2013a).

Recently, there have been efforts to understand the distribution of bacterial secretion systems, particularly T3SS, looking at metagenomic and whole-genome data sets from different ecosystems. This includes fungal-affected habitats like fungus gardens (galleries) and lignocellulose-enriched composts (Barret et al., 2013a). A strong dominance of T3SSs in such fungus-affected ecosystems (Integrated Microbial Genomes with Microbiome samples from the Joint Genome Institute accessions, IMG/M ID 2199352008; 2032320008; 2032320009) was found, which further corroborates the tenet of a broad impact of this secretion system on BFI in these settings (Barret et al., 2013a). Fierer et al. (2012) also analyzed cross-biome metagenomes of soil microbial communities in a range of ecosystems, finding higher abundances of protein- translocation (membrane transport including T3SS) genes in fungal-infested systems. However, with respect to finer scales, fungal-affected milieus like the mycosphere and mycorrhizosphere have been understudied and so the role of T3SSs in such fungal-interactive environmental settings needs to be considered.

### Evidence from Cultivation-Based Studies

About a decade ago, Rezzonico et al. (2004) found that the T3SS *hrc*N (syn. *sct*N) gene was present in many biocontrol fluorescent pseudomonads. In fact, these bacteria clustered separately from phytopathogenic proteobacteria in a *hrc*N (syn. *sct*N)-based phylogenetic tree. Later, they reported that the T3SS of the biocontrol *P. fluorescens* strain KD targets the phytopathogen *Pythium ultimum*, promoting cucumber protection (Rezzonico et al., 2005). Inactivation of the T3SS *hrc*V (syn. *sct*V) gene reduced the strain KD biocontrol activity. Furthermore, expression of the *hrc*V (syn. *sct*V) gene in strain KD was strongly stimulated by the presence of *Pythium*, indicating a target-induced activation system. This was not the case for the cucumber plant (Rezzonico et al., 2005).

At the same time, Mazurier et al. (2004) assessed the distribution of the T3SS *hrc*RST (syn. *sct*RST) genes in saprophytic fluorescent pseudomonads and found these to be enriched in the rhizosphere as compared to corresponding bulk soil. A considerable fraction, 35–52%, of the strains was positive for the *hrc*RST (syn. *sct*RST) genes in the rhizosphere, as compared to 22–39% in the bulk soil. The rhizospheres, from which these *hrcRST* (syn. *sct*RST) positive strains originated, might have included mycorrhizospheres. Moreover, T3SS^+^ pseudomonads, belonging to the *P. fluorescens* phylogenetic group, were more abundant in mycorrhizal than in non-mycorrhizal roots of *Medicago truncatula*, and in bulk soil (Viollet et al., 2011). Taken together, these observations suggest that T3SSs are implicated in the interactions between fluorescent pseudomonads, AM fungi and *Medicago* roots in the rhizosphere. This is further supported by the recent demonstration of the contribution of the T3SS to the mycorrhization assistance given in soil by the MHB *P. fluorescens* C7R12 (Pivato et al., 2009). A T3SS- mutant was used here (Viollet et al., 2016). Similar findings were earlier reported for pseudomonads and ectomycorrhizal fungi (Cusano et al., 2011). Interestingly, *P. fluorescens* BS053, a representative of a major bacterial group inhabiting the mycosphere of the ectomycorrhizal fungus *L. proxima*, was positive for *hrc*R (syn. *sct*R), which was used as a marker for the T3SS (Warmink and van Elsas, 2008). In addition, a significant enhancement of the incidence of culturable T3SS-positive bacteria was found in this mycosphere as compared with the respective bulk soil (Warmink and van Elsas, 2008). Specifically, T3SS-containing bacterial species made up 13.4% of cultured isolates from the mycosphere of *L. proxima*, whereas this was only about 2% in bulk soil. Later work reported that all bacteria migrating through soil with the hyphal front of the saprotrophic fungus *Lyophyllum* sp. strain Karsten were positive for the T3SS (Warmink and van Elsas, 2009; Nazir et al., 2012). Hence, it was hypothesized that the T3SS aids in the bacterial migratory response to an emerging mycosphere (Yang et al., 2016). Migration via fungal hyphae using flagellar movement and assistance by attachment via the T3SS may be involved in the probably complex mechanism, which may further include bacterial growth (Zhang et al., 2014; Yang et al., 2016). Warmink and van Elsas (2009) and, later, Haq et al. (2014) proposed a model in which, minimally, flagella-mediated bacterial motility and T3SS-supported attachment are required, next to growth, for successful biofilm formation along the growing fungal hyphae. This presumably complex process was cogitated to encompass a suite of bacterial activities that take place in a sequential process leading to full colonization of the fungal surface.

On another notice, *Candidatus Glomeribacter gigasporarum* (beta *Proteobacteria*) is an endobacterium of the AM fungal species *Gigaspora margarita.* The endobacterial presence modulated fungal physiology, suggesting that the bacterial absence is perceived by *G. margarita* as a stimulus, activating the expression of genes for stress-responsive proteins (Salvioli et al., 2010). During its interaction with the host fungus, the endobacterium expresses type III (next to type II) secretion systems, which may contribute to the host’s ecological fitness (Ghignone et al., 2012). Another betaproteobacterium, i.e., *Mycoavidus cysteinexigens* gen. nov., sp. nov., strain B1-EBT, of the *Burkholderiaceae* (Ohshima et al., 2016), endosymbiotic in the fungus *Mortierella elongata*, was also reported to possess T3SS genes (Fujimura et al., 2014). The latter may play a crucial role in the bacterial invasion of fungal mycelia. We conclude from these collective data that both fungal-adhering and endomycotic bacteria utilize their T3SS to interact with their fungal host to varying avails (**Figure 1B**).

### Indirect Evidence of Involvement of T3SSs in Bacterial–Fungal Interactions

Most of the indirect evidence is based on either or both *frequency-of-occurrence* and *mechanistic* data. Thus, the fungus *Candida albicans*, when infecting nematodes together with *S. enterica* serovar Typhimurium, was found to be inhibited in its filamentation, and a bacterially secreted molecule was implicated in this inhibition (Tampakakis et al., 2009). In another study, *P. aeruginosa* suppressed proliferation of, and killed, *Aspergilus fumigatus*, involving contact-mediated as well as soluble bacterial factors in hyphal killing (Manavathu et al., 2014). In such co-cultures, localized points of hyphal lysis were observed, suggesting bacterially mediated cell wall lysis (Brand et al., 2008). As the T3SS is commonly functionally present in *P. aeruginosa* and *S. enterica* serovar Typhimurium strains (Sturm et al., 2011), we surmised an involvement of this system, much like in other hyphal killing processes (Kim and Mylonakis, 2011). Furthermore, Hoffman and Arnold (2010) observed viable *Proteobacteria* within the hyphae of endophytic ascomycetous fungi, some of which were closely related to *B. rhizoxinica* and *Candidatus Glomeribacter gigasporarum*. Given the fact that the latter two bacterial groups utilize their T3SSs in the interaction with fungal hosts (Lackner et al., 2011a,b; Ghignone et al., 2012), these endomycotic strains might be similar in their usage of the T3SS to inhabit the fungal interior. Finally, cultivation of *Scutellinia scutellata* was not possible without the presence of *Acidovorax*-like cells (Giordano et al., 2013). As *Acidovorax* species are known to have functional T3SS (Kondo et al., 2012), we here posit a key role for these species and their T3SSs.

Although T3SSs thus indeed appear to mediate a suite of bacterial–fungal interactions, one should keep in mind that, in addition to other bacterial traits including bacterial metabolites, other secretion systems are known to also contribute to BFI (Frey-Klett et al., 2011; Scherlach et al., 2013; Moebius et al., 2014).

### Ecological Effects and Mechanisms of T3SS-Mediated BFI

The T3SS-mediated BFI may involve different mechanistic strategies. In this respect, Kim and Mylonakis (2011) described the effect of the *S. enterica* T3SS on *C. albicans* (**Table 1**). The interaction was presumably mediated by the *sop*B (*Salmonella* outer protein B) gene product, a T3SS-secreted effector molecule (Kim and Mylonakis, 2011). Deleting the *sop*B gene (which encodes inositol phosphatase) significantly decreased the killing of *C. albicans*, similar to that caused by the deletion of *sip*B (*Salmonella* invasin protein) (which encodes T3SS translocation machinery components) (McGhie et al., 2002). Translocation of the *sop*B product to the fungal filaments was found to occur through *sip*B during coinfection, because no signal was observed for *sop*B translocation when the *sip*B mutant was used (immunodetection assay). Moreover, *C. albicans* supernatants were found to upregulate the *S. Typhimurium sop*B and *sip*B genes. Interestingly, the *sop*B gene product negatively regulated the transcription of the *CDC42* gene, which is involved in ‘maintenance of fungal viability’ (Kim and Mylonakis, 2011). Moreover, *sop*B or *sip*B deletion strongly decreased bacterial attachment to *C. albicans* filaments, which was abolished by complementation of *sop*B (Kim and Mylonakis, 2011).

Considering the impact of T3SSs on BFI, an effect was found in the biocontrol agent *P. fluorescens* Pf29Arp. This organism reduces the severity of *Gaeumannomyces graminis* var. *tritici* (*Ggt*) incited take-all disease in wheat. It harbors, along with a T6SS, a T3SS. The T3SS genes were differentially expressed on Ggt-affected (necrotic) versus healthy roots, which suggests that pathogenicity is induced by the T3SS, influencing the lifestyle of strain Pf29Arp in fungal-infested root environments (Marchi et al., 2013). Moreover, T3SSs may play roles in symbiotic interactions of bacteria/fungi and plant roots rather than only with fungi. The mycorrhization helper bacterium (MHB) *P. fluorescens* BBc6R8 has been reported to promote the ectomycorrhizal symbiosis between *Laccaria bicolor* and *Douglas* fir roots (Cusano et al., 2011). In the draft genome of strain BBc6R8, a T3SS was identified which was similar to the one of biocontrol strain *P. fluorescens* SBW25. BBc6R8 T3SS mutants did not affect the radial growth rate of *L. bicolor*, as compared to the wild-type strain. However, they were unable to promote plant mycorrhization by this host fungus, and so the T3SS was implied as a key factor (**Table 1**) in the mycorrhization helper effect (Cusano et al., 2011). Similarly, using a T3SS- mutant, Viollet et al. (2016) have shown that T3SS functioning is involved in the mycorrhization of *M. truncatula* with indigenous AMF by *P. fluorescens* C7R12, whereas the effect of both strains on AM growth in the absence of a plant did not differ. Moreover, T3SS functioning could also impact symbiosis via modifications of the microbiomes of the mycorrhizosphere (Viollet et al., 2016).

In addition, bacterial T3SSs can promote endomycotic life, as modulators of host cell physiological activities. The obligate endosymbiotic *B. rhizoxinica* is found to be very closely associated with its fungal host *R. microsporus.* This organism represents a remarkable and prominent fungal-interactive *Burkholderia* type. When endomycotically present, *B. rhizoxinica* incites the production of toxins, i.e., ‘rhizoxin’ and ‘rhizonin.’ Moreover, it significantly hampers spore formation and consequently controls the reproduction of *R. microsporus*, making the BFI very tight (Partida-Martinez et al., 2007). Valdivia and Heitman (2007) hypothesized that particular effector proteins of *B. rhizoxinica* translocated by the T3SS affect the fungal host. Later on, Lackner et al. (2011a) stated that *B. rhizoxinica* thus controls host reproduction rate (**Table 1**). T3SS defective mutants (*sct*C and *sct*T) exhibited reduced intracellular survival and also failed to elicit sporulation of the host. Moreover, several T3SS genes were upregulated during the cocultivation of *B. rhizoxinica* and host *R. microsporus* (Lackner et al., 2011a).

Another example of a close bacterial–fungal association is *B. terrae* interacting with the saprotrophic fungus *Lyophyllum* sp. strain Karsten. Different fungal-interactive *Burkholderia* strains were found to harbor T3SSs (Warmink and van Elsas, 2009; Nazir et al., 2012, 2013b; Nelson and Sadowsky, 2015). Whole genome sequencing of these bacteria demonstrated the presence of at least one T3SS in the mycospheric *Burkholderia* strains (Nazir et al., 2013b). Very recently, a knock-out mutant of fungal-interactive *B. terrae* BS001 for the *sct*D gene (basal plate gene for T3SS) was constructed, which showed no significant difference to the wild-type strain for growth and nutrient utilization. The migration ability of the Δ*sct*D mutant along with growing hyphae of *Lyophyllum* sp. strain Karsten and *T. asperellum* 302 was hampered, as compared to that of the wild-type. Noticeably, such migration impairment was observed only in mixed-inoculation (i.e., wild-type and mutant coinoculation) experiments (Yang et al., 2016). Then, the adherence of *B. terrae* BS001 to *Lyophyllum* sp. strain Karsten was also evaluated by comparing the Δ*sct*D and wild-type strains. Adherence was reduced for BS001-Δ*sct*D, but conditions under which this effect was dominant are still being explored (Haq et al., 2016). Thus the T3SS was not essential but rather played a helper role in the interaction of *B. terrae* with the soil fungus *L.* sp. strain Karsten (**Table 1**). Overall, we conclude that the T3SS has been evolutionarily employed in varying manners as an ecologically important cellular device that promotes bacterial fitness in a suite of diverse interactive situations.

### Phylogenetic Analysis of the T3SSs of Fungal–Interactive Bacteria

**FIGURE 2:**
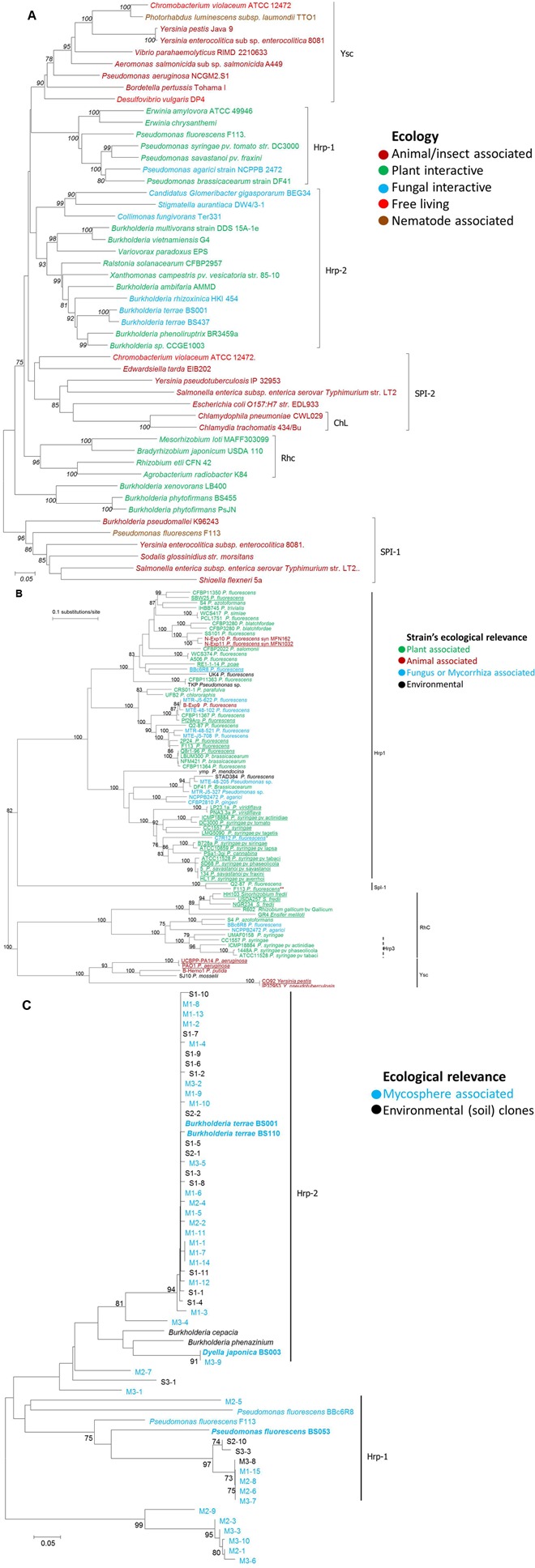
**Phylogenetic analysis of different type III secretion systems (T3SS).** Trees were based on **(A)**, *sct*RST **(B)**, and **(C)** gene respectively, including sequences from fungal-interactive bacterial strains and constructed via neighbor joining method considering Maximum Composite Likelihood model (Jukes and Cantor for **B**) with Bootstrap replicates of 1000, and bootstrap values equal to or greater than 70% are shown. Different font colors: ecological role or relevance of respective bacterial hosts. **(B)** Underlined strains with T3SS role described. ^∗^Strains reported to interact with fungal hosts via their T3SS. ^∗∗^One (of two) T3SS copy (of plant-associated strain) reported to mediate interaction with nematodes. **(C)** adapted from Warmink Warmink and van Elsas (2008); **bold** sequences from isolates, others: sequences from clones or references. M, mycosphere-derived; S, bulk soil derived. Codes, respective samples.

Phylogenetic analysis of bacterial T3SSs based on the *sct*N gene (SctN is a T3SS-encoded ATPase; syn. HrcN, YscN, EscN, InvC, SsaN) exhibited different T3SS clusters. Noticeably, ecological relevance of the respective host bacteria may be connected with the T3SS type present in them (**Figure 2A**). For instance, animal pathogens have Ysc and SPI-1 types; The ChL and SPI-2 types are present in another bifurcation, including Chlamydiales and protist-interactive/free living bacteria. On the other hand, plant-interactive bacteria are mainly distributed in the Hrp-1, Hrp-2 and RhC T3SS clusters. More interestingly, this analysis demonstrated that all fungal–interactive bacterial T3SSs are clustered into the Hrp2 family (**Figure 2A**). Thus, on the basis of the T3SS, there seems to be a restricted evolutionary path among bacteria toward ‘fungal interactivity.’ This tenet is thought to hold at least for those bacteria that ‘learned’ to employ a T3SS in their (evolutionarily successful) interactivity with host organisms. In detail, the mycolytic *C. fungivorans* Ter331 and the wood rot bacterium *Stigmatella aurantiaca* DW4/3-1 constitute a small divergent subgroup from the main cluster, along with the endofungal *Candidatus Glomeribacter gigasporarum BEG34*. Another strongly fungal-interactive organism, the endomycotic *B*. *rhizoxinica* HKI 454 (host: *R. microsporus*), makes part of the main cluster close to that formed by *Lyophyllum* sp. associated *Burkholderia* strains. Most of the other members of this Hrp2 family are *Grosso modo* rhizosphere inhabitants. We here raise the possibility that these rhizosphere dwellers may gain ecological and evolutionary benefit from their interaction with root-associated fungi, thus suggesting a possible role of their T3SS in such associations. Moreover, the plant-interactive *B. phytofirmans* (originally isolated from surface-sterilized *Glomus vesiculiferum* – infected onion roots) and *B. xenovorans* (normally found in the rhizosphere of grasses and able to fix nitrogen) form a distinct group separate from the seven T3SS families described. This may lead toward a new class of rhizosphere-associated *Burkholderia* type of interactive T3SS.

Another phylogenetic tree – constructed on the basis of pseudomonad *sct*RST (syn. *hrc*RST, *psc*RST, *rsc*RST, *rhc*RST) gene sequences – clearly shows that a great diversity of such sequences are grouped within the Hrp1 T3SS family, which probably includes at least three subfamilies (**Figure 2B**). It is interesting to note that this very diverse family encompasses sequences from strains which have been shown to interact with eukaryotic hosts belonging to various kingdoms (animals, plants, and fungi) (**Figure 2B**). The *sct*RST tree contains numerous biocontrol pseudomonad strains, many of which harbor T3SSs (Loper et al., 2012); some of them, which exhibit antagonistic activities against pathogenic fungi, have also been associated with the presence of T3SSs. The Hrp1 T3SS family contains all pseudomonad strains isolated from fungal environments and/or enriched in the presence of mycorrhiza or mycorrizal fungi. These include strains BBc6R8 and C7R12. For both strains, the importance of the T3SS on the MHB effect has been demonstrated using T3SS- mutants, using ectomycorrhiza and arbuscular endomycorrhiza, respectively (Cusano et al., 2011; Viollet et al., 2016).

Since *sct*RST genes as such are absent from the Hrp2 family, an additional phylogenetic analysis of *sct*R sequences (syn. *hrc*R, *ysc*R, *spa*P, *ssa*R, *spa*24), present in both the Hrp1- and Hrp2-T3SS families, was performed on a set of sequences obtained from the *L. proxima* mushroom foot (Warmink and van Elsas, 2008). A majority of the fungal-interactive *sct*R sequences grouped in the Hrp2-type T3SS family, along with *B. terrae.* However, some, including mycospheric *P. fluorescens* BS053, were part of the Hrp1 type (**Figure 2C**). This confirms the importance of these two T3SS families in BFI.

Altogether, the phylogenetic analyses based on *sct*N, *sct*RST and *sct*R indicate that the fungal–interactive bacterial T3SSs are mainly part of the Hrp1 and Hrp2 T3SS families. More precisely, it appears that the fungal–interactive pseudomonads belong to the Hrp1 T3SS family, while the other fungal–interactive bacteria belong to the Hrp2 family. Overall, Hrp-positive bacteria can be enriched in the rhizosphere (Mazurier et al., 2004) and mycorrhizosphere of different plant species (Warmink and van Elsas, 2008; Viollet et al., 2011), and so we surmised that T3SSs are often involved in BFI which may include plants (BF-plant interactions – BFPI).

## What Did We Learn? – Conclusion and Perspectives

We here provide evidence for the contention that bacterial T3SSs (particularly of the Hrp1 and Hrp2 families) are often involved in interaction processes of bacteria with fungi in soil and plant habitats, as well as with other eukaryotic organisms. Overall, T3SSs constitute fascinating trans-kingdom communication devices, which allow bacterial cells to adhere to the surfaces of eukaryotic cells and inject proteins or other effectors, in order to obtain an ecological advantage by destroying or subverting the target cell (Cornelis, 2006). T3SSs, thus, in a generic sense, enhance the provision of nutrients from a target organism to the interactive T3SS-endowed bacterium. However, they might also act as adherence devices that allow the T3SS-carrying bacteria to obtain an ecological edge by assisting in the co-migration with host fungi by adhering to the host cells, allowing an enhancement of occupancy of a local niche. Very speculatively, they might endow their host cells with the capacity to build a better biofilm at the fungus, much like shown for *Erwinia chrysanthemi* (Yap et al., 2005).

We examined different lines of evidence with respect to the contribution of the T3SS to different ecological outcomes of the interactions of their host bacteria with fungi (Warmink and van Elsas, 2008, 2009; Cusano et al., 2011; Viollet et al., 2011, 2016; Nazir et al., 2012; Haq et al., 2016; Yang et al., 2016). First, the robust evidence for an involvement of the T3SS in the interaction of *B. rhizoxinica* with *R. microsporus* in rice (Lackner et al., 2011a) unequivocally revealed the key role of this system in the modulation of fungal physiology (sporulation). Along the same line, Nazir (2012) reported that the T3SS of *B. terrae* BS001 was highly expressed in liquid microcosms where mushroom formation of *L.* sp. Karsten was inhibited and glycerol release stimulated (Nazir, 2012). An involvement of this secretion system in such processes was therefore suggested, but the hypothesis still requires stronger evidence. The mycorrhization helper effect of given model strains of pseudomonads was also shown to be related to their T3SSs (Cusano et al., 2011; Viollet et al., 2016). However, we still do not understand whether such T3SS-based modulations of host physiologies take place by translocation of similar or different effectors and what critical factors play roles here.

With respect to the T3SS acting as an adherence device, Yang et al. (2016) recently provided evidence for the tenet that T3SS^+^ *B. terrae* cells are more avid co-migrators with soil fungi than T3SS- counterparts. The positive effect of the T3SS on co-migration was attributed to a helper effect, which was, however, rather weak. Indeed, the T3SS- cells could still co-migrate with the moving hyphal front through soil, albeit to a reduced extent. Thus, co-migration with soil-exploring fungi was spurred by the T3SS, yet there was no absolute dependency. This observation highlights that BFI are not only mediated by T3SSs and that other mechanisms may also contribute to these interactions.

Type III secretion system-positive bacteria may also affect the physiology of fungal hyphae by acting at their surface, e.g., by restricting or stimulating fruiting body formation (Nazir, 2012). Another possible effect (corollary) of active T3SSs might be the shutting down of fungal defense mechanisms against bacteria (Wohlschlager et al., 2014; Kim et al., 2015). In this way, the fungal-associated bacteria would create their own microhabitat and intimate interaction at the surface of fungal hyphae. At the fungal surface, T3SS-positive bacteria form biofilms which may become prone to grazing by soil protozoa. A putative role for the T3SS in both biofilm formation and protection from protozoal grazing may be postulated on the basis of the still sparse evidence contained herein (Kim and Mylonakis, 2011; Jennings et al., 2012). However, definite proof for these hypotheses has yet to be found and landmark studies on the role of T3SSs in the interactions of specific soil bacteria with fungi are urgently needed. In particular, the putative roles of the T3SS in (1) survival at the fungal surface in the presence of predating protozoa, and (2) biofilm formation at the surface, are intriguing. The T3SS-encoded cellular appendices might play roles in (1) the formation of biofilms at the fungal surface (Warmink and van Elsas, 2009), (2) the enhancement of adherence to such surfaces, (3) the formation of cell clumps and (4) generally the stimulation of aggregative processes. Such hypotheses provide interesting leads that may guide further investigations of the underlying ecological processes. The resulting biofilms might assist the host populations in ecological settings, e.g., providing protection against protozoan grazing and/or antimicrobials. Such concepts are supported by scientific data (Jennings et al., 2012; Nazir et al., 2014; Haq et al., 2017), but mechanistic studies are still required in this domain. Therefore, future work should focus on the role of the T3SS in biofilm formation and maintenance on mycelial networks in ecological settings. Moreover, the overwhelming knowledge on pseudomonads interacting (as pathogens) with fungi (Scherlach et al., 2013), and also the presence of T3SSs across these pseudomonads (Mazurier et al., 2015), demand a special focus to be placed on the possible connection between such findings.

Research on secretion systems is a moving field of science with increasing evidence of the contribution of T3SSs in BFI, including beneficial but also deleterious ones. The demonstration that T3SSs are not only involved in pathogenesis between bacteria and eukaryotic organisms but also in synergistic effects clearly widens the role of these secretion systems. A better knowledge of the mechanisms that underlie the effects, including the identification and target of the effector molecules, would open further prospects for using these secretion systems in order to modulate bacterial–fungal interactions to benefit plant growth and health. In a generic sense, novel studies on the functioning of the T3SS during bacterial–fungal associations will yield strongly improved scientific insights about the mechanisms these microorganisms use in the interactions in order to achieve ecophysiological and evolutionary success. Consequently, the improved knowledge would enable their utilization in more efficient way in different environmental settings for the improvement of sustainable ecosystems. Thus, it is hoped that this synthesis may help to validate this concept in order to foster the use of bacterial–fungal consortia in different domains of life.

## Author Contributions

RN, PL and JvE initiated the concept; SM prepared a phylogenetic tree, RN prepared figures and table and all three drafted the manuscript; JvE, SM, PL revised the manuscript to improve it while PY helped meanwhile.

## Conflict of Interest Statement

The authors declare that the research was conducted in the absence of any commercial or financial relationships that could be construed as a potential conflict of interest.
